# Therapists’ Experiences With the Effectiveness and Feasibility of Videoconference-Based Eye Movement Desensitization and Reprocessing

**DOI:** 10.3389/fpsyg.2021.748712

**Published:** 2021-10-05

**Authors:** Corinna Mischler, Arne Hofmann, Alexander Behnke, Lynn Matits, Maria Lehnung, Suchithra Varadarajan, Roberto Rojas, Iris-Tatjana Kolassa, Visal Tumani

**Affiliations:** ^1^Department of Psychiatry, Ulm University Hospital, Ulm, Germany; ^2^Clinical & Biological Psychology, Institute of Psychology and Education, Ulm, Germany; ^3^EMDR-Institute Germany, Gezeitenhaus Traumahospital Schloss Eichholz, Wesseling, Germany; ^4^EMDR-Institute Germany, Private Practice, Eckernfoerde, Germany; ^5^University Psychotherapeutic Outpatient Clinic, Institute of Psychology and Education, Ulm University, Ulm, Germany

**Keywords:** eEMDR, videoconference-based EMDR, EMDR-online, therapist experiences, traumatherapy in pandemic, internet-based trauma-therapy, trauma-focused psychotherapy, COVID-19 pandemic

## Abstract

Research on the effectiveness and applicability of eye movement desensitization and reprocessing (EMDR) *via* videoconference is sparse. Considering the emerging use of internet-based psychotherapy during the COVID-19 pandemic, information on videoconference-based EMDR (eEMDR) would be beneficial for many therapists. In this study, 23 therapists from the EMDR-Institute in Germany provided information about their experiences with eEMDR in a questionnaire-based survey. Information on the effectiveness and the course of 102 eEMDR sessions was recorded. Results showed the potential of eEMDR as an effective and viable method. The decrease in the subjective unit of disturbance (SUD), which is an important indicator of treatment outcome, was found to be at a similar level compared to that of previous EMDR studies that were not administered in eEMDR format. The most important predictor of the SUD decrease was the type of bilateral stimulation used in eEMDR sessions. Eye movements resulted in significantly greater SUD reductions than tapping. Perceived disadvantages and impediments for the implementation of eEMDR were mainly of bureaucratic and technical concerns. In addition, about one-third of the therapists stated that some patients were not willing to engage in eEMDR. In our study, eEMDR proved to be a practically applicable therapy method and therefore, therapists can consider using eEMDR. These findings will hopefully encourage EMDR therapists and their patients to use eEMDR due to its effectiveness and viability as an online treatment approach.

## Introduction

Mental health consequences of the COVID-19 pandemic suggest a high demand for psychological support during this unprecedented situation (e.g., Benke et al., 2020). At the same time, therapists are constrained from conducting face-to-face psychotherapy sessions due to the risk of infection. To resolve this challenge, an increasing number of therapists is adapting to internet-based psychotherapy sessions (Békés and Aafjes-van Doorn, 2020; DPtV, 2020).

Eye movement desensitization and reprocessing (EMDR) as well as trauma-focused Cognitive-behavioral therapy (CBT) are the primary choice for treatment of PTSD (WHO, 2013; NICE, 2018). EMDR is an eight-phase approach for the treatment of PTSD, developed by Dr. Francine Shapiro in 1987 (Shapiro, 1996; Hofmann, 2014). Both EMDR and CBT are efficacious in treating PTSD symptoms in face-to-face settings (Seidler and Wagner, 2006). Moreover, there is ample evidence for the efficacy of internet-based CBT in the treatment of PTSD symptoms (Knaevelsrud and Maercker, 2007; Lewis et al., 2019). Thereby, internet-based therapy includes both synchronous and asynchronous formats. Synchronous formats mostly cover videoconference-based therapy sessions with direct contact with a therapist, whereas asynchronous formats include different interventions and trainings that are intended to be mainly self-directed (Kuhn and Owen, 2020).

A recently published review showed a lack of available evidence on effects of internet-based EMDR (Lenferink et al., 2020). Furthermore, there are doubts whether the implementation of EMDR *via* videoconference is feasible and appropriate (Gibson et al., 2009; DPtV, 2020). As of now, there are only three studies which examined the effectiveness of internet-based EMDR (Todder and Kaplan, 2007; Spence et al., 2013; Tarquinio et al., 2021). In the study of Tarquinio et al. (2021) an adapted EMDR protocol was applied. Healthcare providers suffering from the effects of the sanitary crisis in hospitals due to the COVID-19 pandemic were treated with the adapted protocol (URG-EMDR) *via* videoconference. Their distress, measured as a reduction of the subjective unit of disturbance (SUD; Wolpe, 1969), significantly decreased in one session. In a study of Spence et al. (2013) patients received a six-lesson online intervention with a combined treatment protocol, i.e., trauma-focused CBT (TF-CBT) and EMDR with a web-based EMDR tool. Results showed a decline in PTSD symptoms both directly after the intervention and after 3months. A single-case report, Todder and Kaplan (2007) described the successful treatment of a patient with a traumatic memory through a single EMDR session conducted *via* videoconference. These promising findings illustrate the potential of videoconference-based EMDR (“eEMDR”); however, they do not provide general conclusions about the effectiveness of eEMDR in an outpatient treatment setting. In times of COVID-19 and beyond, improvements in the provision of eEMDR and information on its effectiveness in treating PTSD especially in routine outpatient care are strongly required.

To address this research gap, we studied the effectiveness of eEMDR on patients with PTSD and other diagnoses within the framework of an explorative approach. For the study purpose, we consulted therapists from the EMDR-Institute in Germany regarding their experiences with eEMDR sessions during the COVID-19 pandemic. Our study focused on obtaining information regarding the effectiveness of eEMDR sessions in a standard outpatient routine treatment, i.e., ratings of the EMDR process and the SUD decrease in patients reported during an eEMDR session. Notably, the SUD decrease can be seen as an important tool for therapists to assess the treatment process (Kim et al., 2008). Potential moderators of these indicators of effectiveness were considered; we particularly examined possible influences of different modes of bilateral stimulation and the therapists’ professional experience on the treatment outcome. Previous research on the latter topic showed inconsistent results (Propst et al., 1994; Tschuschke et al., 2015; Goldberg et al., 2016). Furthermore, we collected information on the technical aspects of eEMDR sessions and challenges in implementing eEMDR. In addition, we also collected therapists’ qualitative feedback concerning perceived advantages and disadvantages as well as required improvements of eEMDR sessions. Overall, our study aimed to provide explorative information on the feasibility and effectiveness of eEMDR.

## Materials and Methods

### Participants

#### Therapists

Four hundred EMDR-therapists were addressed *via* email, and 32 (8%) of them responded and returned filled-in questionnaires. Therapists were mainly female (81.3%) and more than half of them (65.6%) were aged between 51 and 65years. They had a mean professional experience of 19.77years (*SD*=9.23, range=4–35years) and a mean EMDR experience of 10.72years (*SD*=6.59, range=1–28years). Therapists were mainly trained in administering CBT (56.3%) and depth psychology (40.6%). Some therapists (12.5%) indicated they have already had experience with online-based psychotherapy before the COVID-19 pandemic.

#### Patients

**Figure 1:**
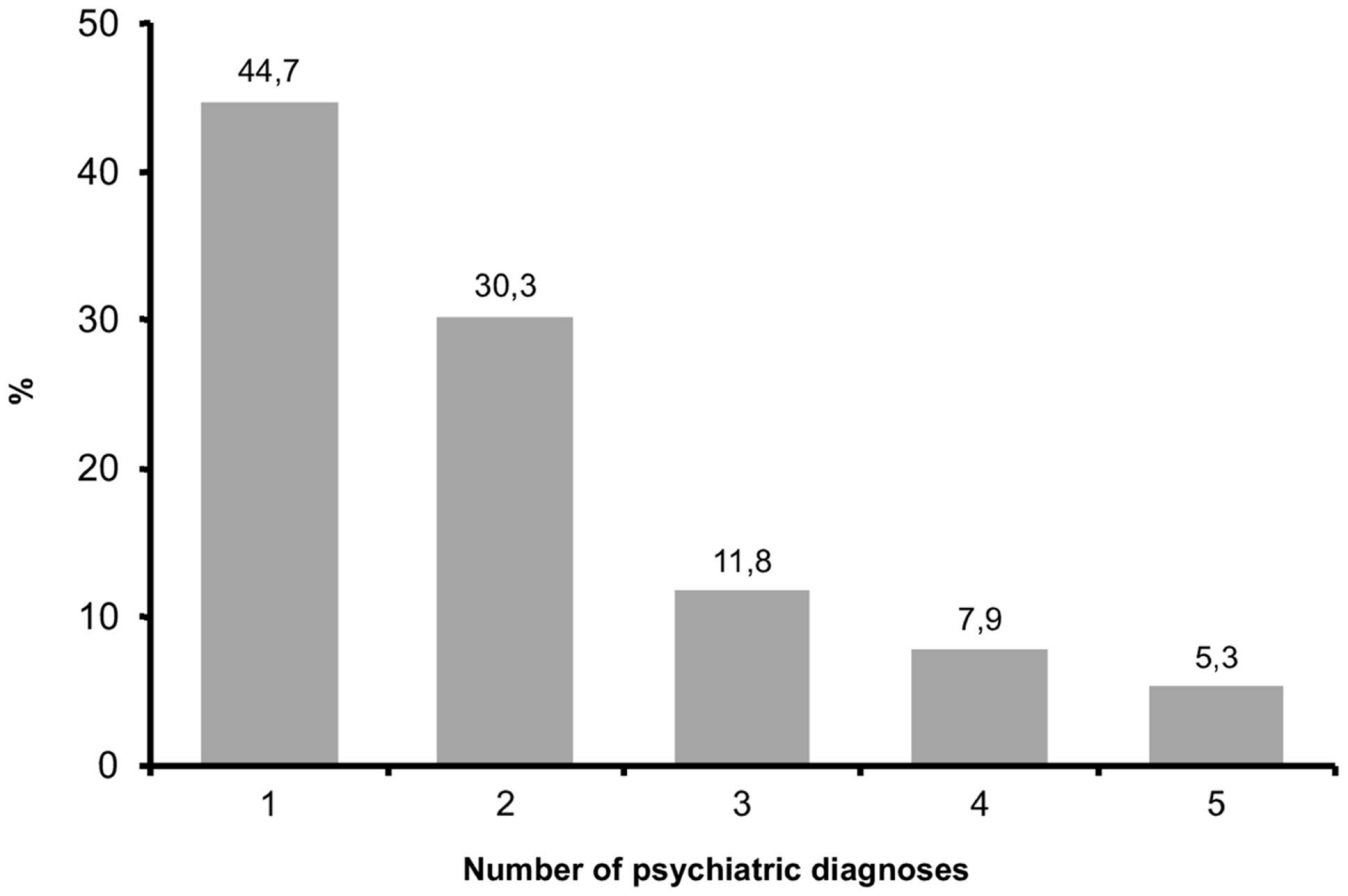
Number of patients’ psychiatric diagnoses (*N*=76).

Therapists provided session data for a total of 76 different patients. Patients were mainly female (77.6%) and aged between 18 and 68years (*M*=41, *SD*=12.09). On average, patients had received 4.84 eEMDR sessions (*SD*=5.28, range=0–30). Regarding their mental health, roughly 50% of the patient cohort fulfilled criteria of one diagnosis, while the other half of the patients had multiple comorbid diagnosis (see Figure 1).

**Figure 2:**
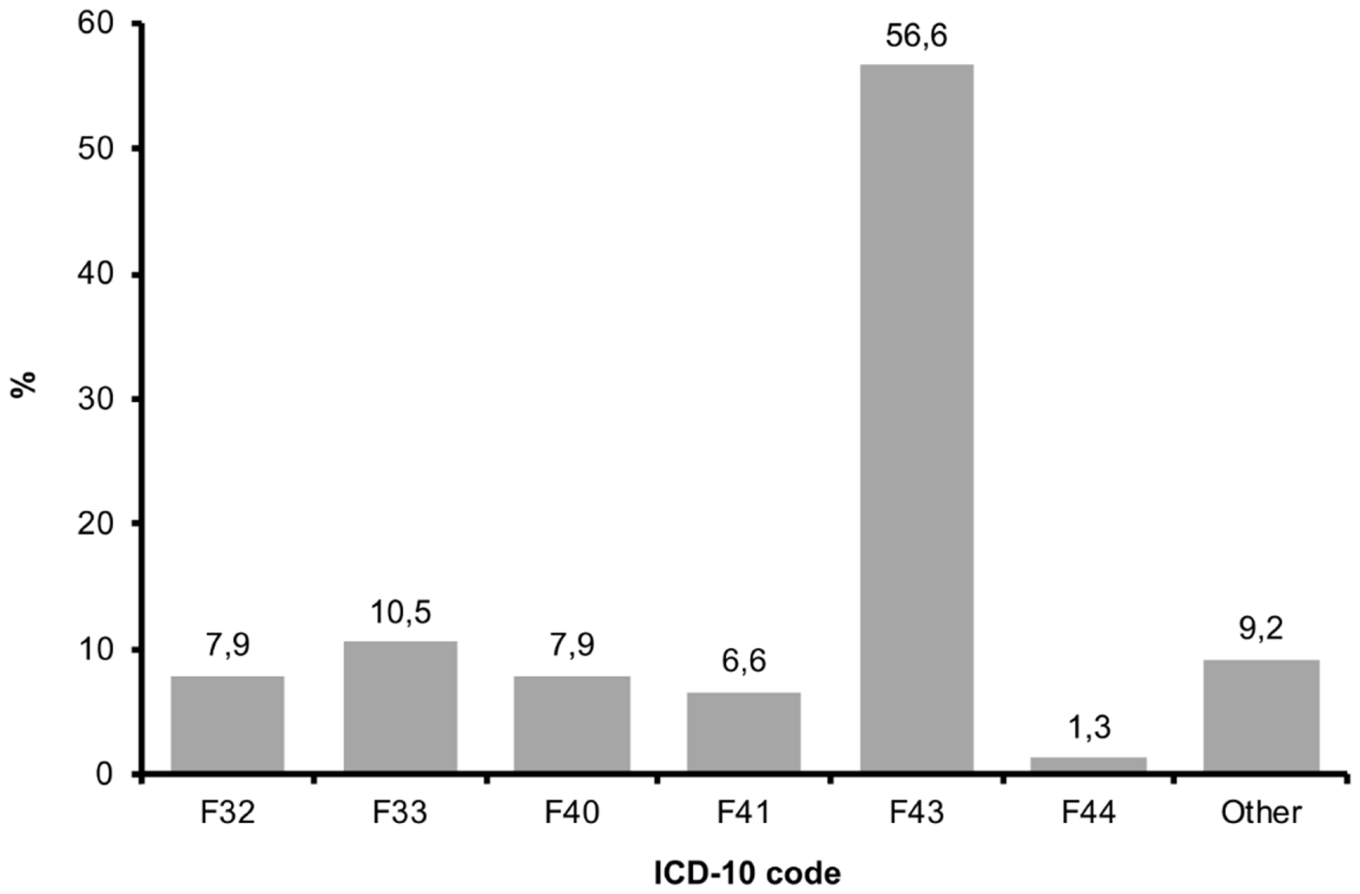
Patients’ main diagnosis grouped after ICD-10 code (*N*=76).

Figure 2 shows patients’ main diagnoses grouped according to ICD-10 code. The majority of patients were diagnosed with a disorder from cluster F.43 “acute stress reactions.” Secondary diagnoses were mainly psychological problems (i.e., depressive, anxiety, dissociative, somatoform, eating and personality disorders), whereas four patients were also diagnosed with somatic diseases (malignant neoplasm of prostate; obesity due to access calories; 2× relapsing-remittent multiple sclerosis).

#### Sessions

**Figure 3:**
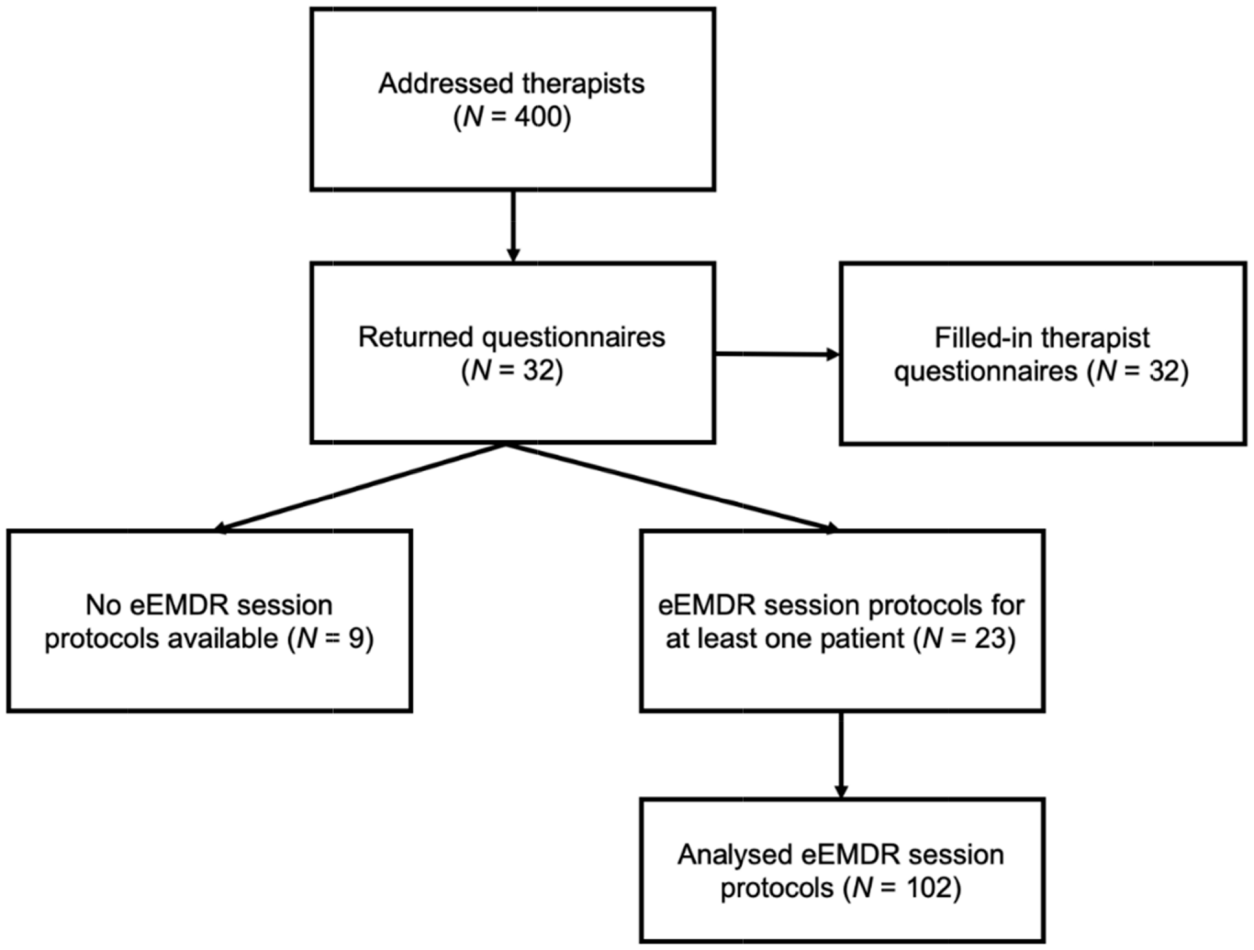
Therapist and session flow chart.

Therapists were requested to return eEMDR session protocols of at least three different patients. Nine therapists did not return protocols because they had not yet conducted eEMDR. However, they provided information on generic questions like perceived barriers to online therapy, alternatives to eEMDR in COVID-19 pandemic, and experience with online therapy in general. The remaining 23 therapists on average returned 4.44 eEMDR session protocols (*SD*=4.50, range=1–22) and eventually, 102 protocols were available for evaluation. Figure 3 shows the flow chart of participating therapists and eEMDR session protocols. Mean duration of eEMDR sessions was 61.21 (*SD*=19.75, range=30–100) minutes.

### Questionnaire

Therapists were sent a survey consisting of two parts, i.e., 21 questions regarding the therapist and 36 questions regarding the eEMDR session, each comprising qualitative and quantitative items and all answers were provided by therapists. Based on the usual procedure, SUD values at the beginning and end of the session were reported by patients.

#### Therapist Module

Therapists reported sociodemographic data, information about their professional background (e.g., “*Since when are you practicing EMDR?*”), their experiences with online-based psychotherapy (e.g., “*Do you have experience with video-based psychotherapy/online therapy in general?*”) and provided description of the technical as well as organizational measures they implemented to administer eEMDR (e.g., “*Which technical equipment did you use for the eEMDR treatment?*”).

#### Session Module

The session-related questions concerned information about the patients (e.g., age, gender, diagnoses), technical aspects of the eEMDR session (e.g., duration, time, use of different protocols, mode of bilateral stimulation, type of eye movements), and ratings concerning the quality of the eEMDR session (e.g., quality ratings of eEMDR sessions compared to face-to-face EMDR sessions, decrease in SUD ratings from beginning of a session to its end, ratings of process, adherence, confrontation, and grounding). Moreover, on qualitative items, the therapists reported their opinion on advantages and disadvantages of eEMDR as well as adjustments and improvements necessary for the further use of eEMDR.

### Procedure

Questionnaires were sent *via* e-mail to therapists from the EMDR network in Germany. The e-mail informed therapists about the purpose and all procedures of the study as well as data protection issues. Most of the questionnaires were returned *via* postal mail; only a few were returned *via* e-mail. All study procedures were approved by the ethics committee of Ulm University.

### Statistical Analysis

Data were prepared and analyzed with IBM SPSS statistics (version 26.0.0.0) and R (version 1.3.1093). Our aim was to relate patient and therapist characteristics to quantified session effectiveness. Decrease in SUD ratings was analyzed for associations with potentially influential variables using Spearman’s rank correlation coefficients and Pearson’s correlation coefficients where applicable. Group comparisons with potential predictors of SUD decrease were computed with Kruskal-Wallis tests. Bonferroni-Holm corrections were conducted to correct the results for multiple testing. A multiple linear regression analysis was computed to examine the combined relevance of various predictors of SUD decrease. Qualitative statements about therapists’ attitudes towards eEMDR were collected as free text. The statements were reviewed, and frequency statistics were derived based on superior categories. This classification was crosschecked by one of the other authors (VT).

## Results

### Technical Aspects of eEMDR

#### Technical Devices

In most eEMDR sessions, patients used computers or laptops (86.8%) followed by smartphones (18.4%). Likewise, therapists conducted the sessions mainly on computers or laptops (95.7%), followed by smartphones (8.7%), and tablets (8.7%). Some therapists also switched between different technical devices. Occasionally, they used additional equipment, i.e., headsets (30.4%), telephones (17.4%), and additional cameras (8.7%).

#### Use of Protocols

Eye movement desensitization and reprocessing is a treatment algorithm developed by Shapiro that uses 8 phases and 3 temporal focusing steps to process stressful material. EMD is a modified EMDR protocol that focuses on a single high charge memory. EMDr is another slightly differently modified EMDR protocol that focuses on a single memory and allows only a limited range of associations in the processing phase (Shapiro, 2013). The standard EMDR protocol was conducted in 61.8% of the sessions, EMD in 2.9%, and EMDr in 3.9% of the sessions. Out of the sessions using the standard EMDR protocol, in 73% of sessions, the standard EMDR protocol was exclusively applied, whereas in 27% of sessions, the standard protocol was combined with other protocols (Table 1 shows the most frequent combinations). When the standard EMDR protocol was used, 53.2% of the sessions ended as complete sessions.

**Table 1:** Most frequent combinations of the standard EMDR protocol in eEMDR sessions.

Combination	Frequency (%)
Future perspective	7 (11.1)
Four-fields-technique	3 (4.8)
Flash-technique, grounding, safe place, light stream technique	3 (4.8)
CIPOS, grounding, safe place, light stream technique	3 (4.8)
EMD, EMDr	2 (3.1)
Grounding, safe place	2 (3.1)
Four-fields-technique, grounding, safe place, light stream technique, four-elements-exercise	2 (3.1)

*EMDR, Eye Movement Desensitization and Reprocessing; CIPOS, Constant Installation of Positive Orientation and Safety; EMD/EMDr, variations of the EMDR standard protocol (EMD=strictly focused shape of EMDR; EMDr=more focused EMDR in its association). Relative numbers referring to the complete use of the standard EMDR protocol, solely and combined (N=63)*.

#### Mode of Bilateral Stimulation

In 52.9% of the sessions, bilateral stimulation was administered *via* eye movements and in 36.3% of the sessions *via* tapping. In 7.8% of the sessions, both modes of bilateral stimulation were combined. When using eye movements for bilateral stimulation, supportive tools (e.g., emdr remote, light bar, eye scan etc.) were often (51.9%) used to evoke eye movements. Less often (31.5%), therapists guided the patients’ eyes with their fingers, while acoustical guidance (9.3%) and instructing the patients to switch their gaze between room corners (5.6%) were rarely used.

#### Stop Signal

In most of the sessions, patient and therapist agreed on raising the hand as a stop signal (43.8%), while in 20.8% of the sessions, patient and therapist agreed on saying “stop,” and in 15.6% of the sessions, both signals were used concomitantly. Rarely, other stop gestures (e.g., shaking the head or standing up) were agreed.

### Measures of Effectiveness

#### Ratings of eEMDR Sessions

**Figure 4:**
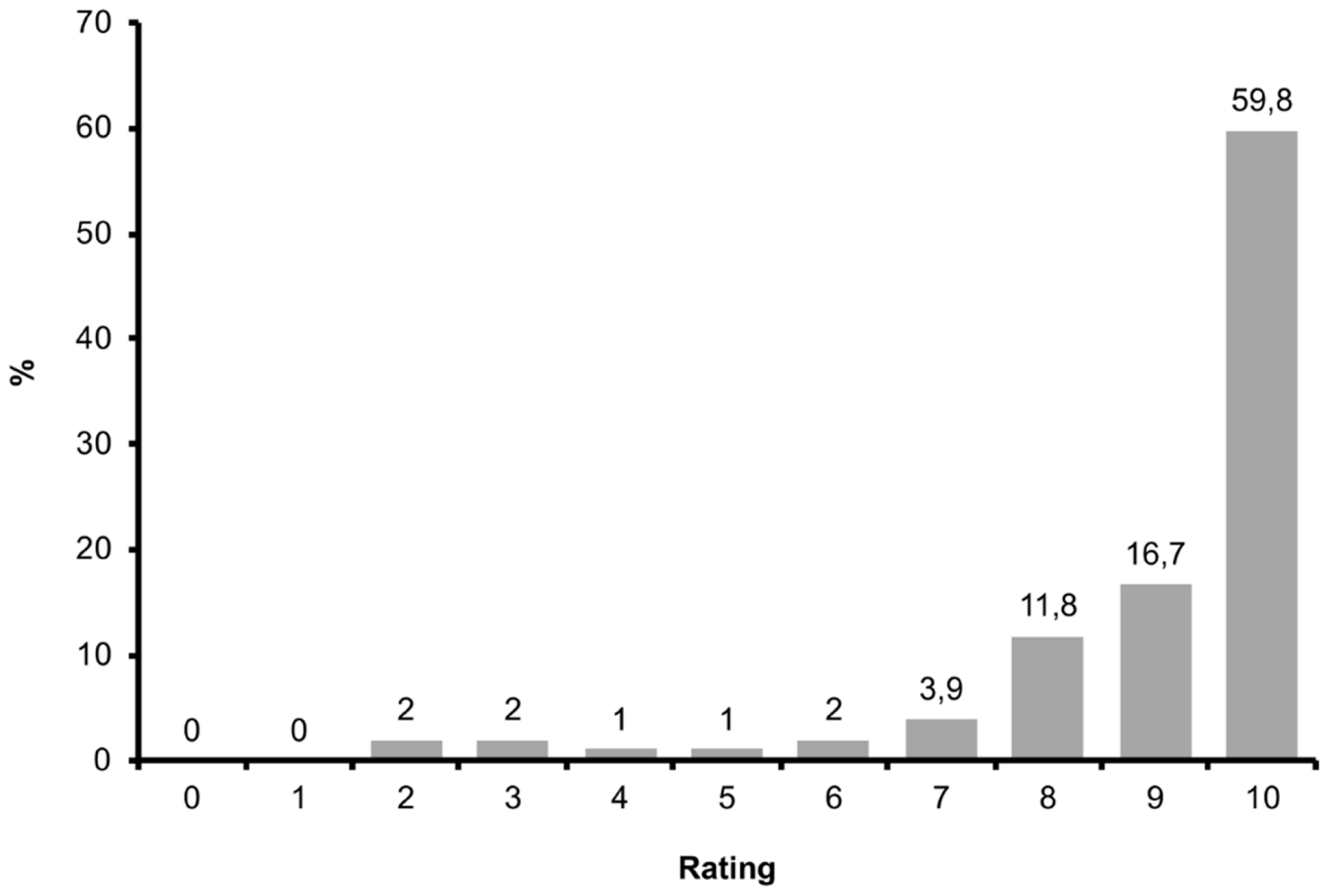
Process ratings in eEMDR sessions (*N*=102). 0=very bad, 10=very good.

In roughly 90% of the sessions, the therapists rated the overall impression of eEMDR sessions as very good (i.e., with eight to ten out of ten possible points). Similarly, when asked more specifically about the impression of the eEMDR process, therapists also submitted very good ratings. Figure 4 depicts the therapists’ process rating in more detail. Medians and means of all eEMDR session ratings are shown in Table 2. Moreover, when compared to face-to-face EMDR, the therapists rated 91.2% of eEMDR sessions as *good* or *very good* (*Mdn*=5 *very good*), and no session has been considered *very bad* as compared to face-to-face EMDR.

**Table 2:** Descriptive statistics of different eEMDR ratings.

Rating	*Mdn*	*M*	*SD*	Range
Overall	10	9	1.77	2, 10
Process	10	8.99	1.98	0, 10
Adherence	10	9.29	1.22	3, 10
Confrontation	10	9.18	1.46	3, 10
Grounding	10	9.19	1.81	0, 10

#### SUD Ratings

On average, the patient-rated SUD decreased by *M*=73.1% from the beginning to the end of an eEMDR session (see Table 3 for details). A relative SUD decrease was calculated to take into account the varying SUD ratings at session begin. As some eEMDR sessions ended incompletely, a relative SUD decrease could not be gained from every session protocol.

**Table 3:** Descriptive statistics of different subjective unit of disturbance (SUD) ratings.

Rating	*Mdn*	*M*	*SD*	Range
SUD beginning	5	7.98	1.6	4, 10
SUD end	2	2.24	2.28	0, 8
SUD phase 8^a^	1	1.83	2.64	0, 10
Relative SUD decrease^b^	77.8	73.1	26.8	11.1, 100

a *Values referring to SUD phase 8 ratings, when sessions were ended as complete sessions; values from incomplete sessions were excluded from this analysis (N=41)*.

b *A relative SUD decrease was calculated to take into account varying SUD ratings at session begin*.

### Influences on SUD Decrease

Correlational analyses indicated significant associations neither between the therapists’ general professional experience and the relative SUD decrease, *r*=0.00, *p*=0.999, nor between the therapists’ EMDR experience and the relative SUD decrease, *r*=−0.02, *p*=0.865. The relative SUD decrease was also independent from patients’ sex, *r*_pb_=−0.02, *p*=0.844, from therapists’ sex, *r*_pb_=−0.13, *p*=0.359, and from patients’ age, *r*=−0.10, *p*=0.404. Depending on the different therapist age categories, there were no differences in the relative SUD decrease either, *χ*^2^(5)=8.82, *p*=0.116. The SUD decrease was lower in sessions with patients who have already had more eEMDR sessions, *r*=−0.23, *p*=0.049.

**Figure 5:**
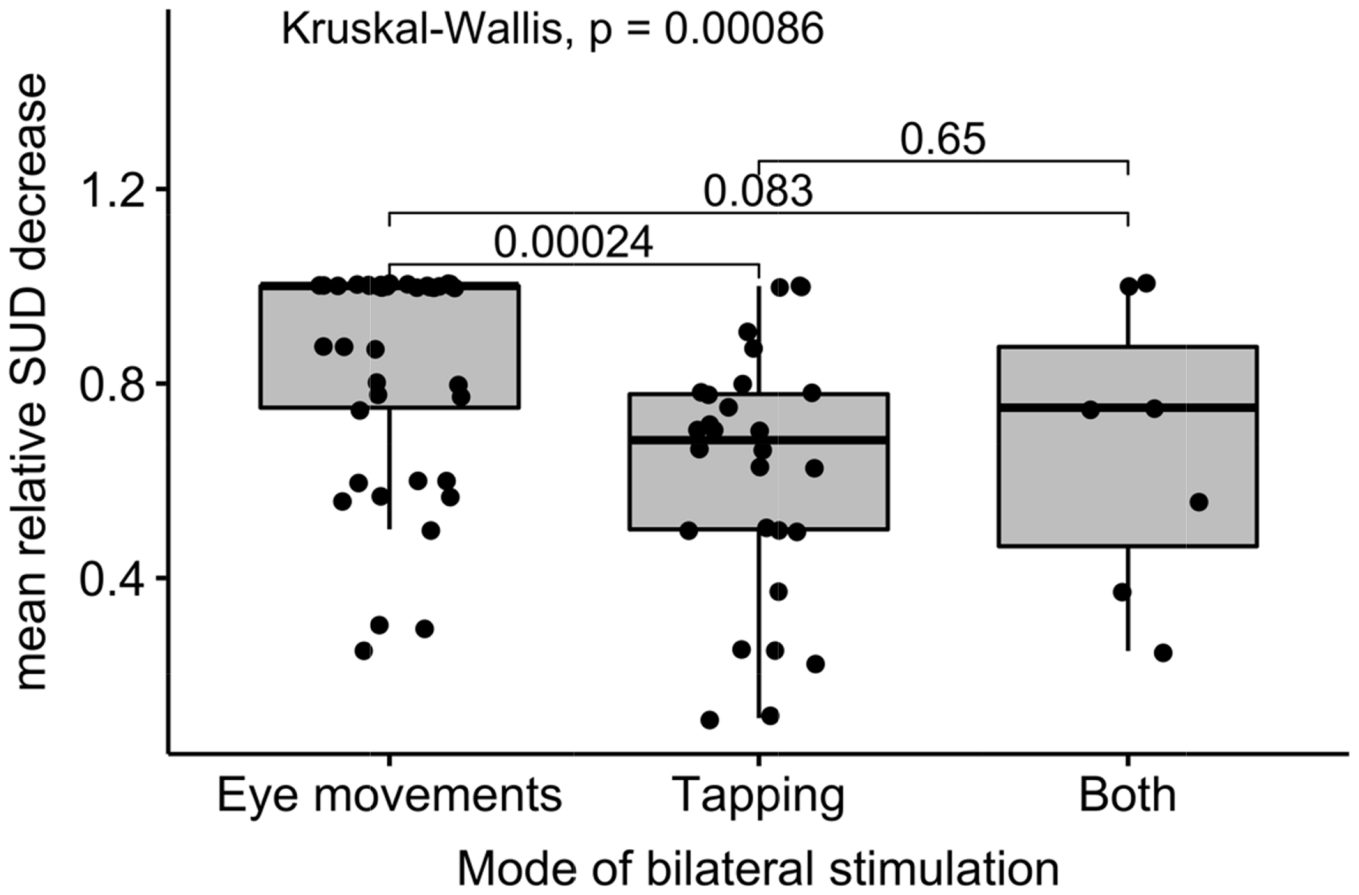
Boxplots for group differences between different modes of stimulation.

The relative SUD decrease differed significantly depending on the mode of bilateral stimulation, *χ*^2^(2)=14.11, *p*<0.001. As displayed in Figure 5, *post hoc* tests showed, eye movements have been more effective in reducing the SUD (*Mdn* =1) than tapping (*Mdn* =0.68).

To test whether the effect of bilateral stimulation still influences the relative SUD decrease when adjusting for the therapists’ general work experience and specific EMDR experience, a multiple linear regression was computed, *F*(4,71)=3.93, *p*=0.006, *R*^2^=18.1%. The analyses corroborated the relevance of the mode of bilateral stimulation as a significant predictor of the relative SUD decrease. However, the therapists’ general work experience and EMDR experience were not of relevance (see Table 4).

**Table 4:** Multiple regression on the association between SUD decrease and the mode of bilateral stimulation, the therapists’ general work experience and their specific EMDR experience.

	*F*	*df*	*B*	*SE B*	95% CI	*p*	$\eta_{p}^{2}$
(Constant)	50.50	1, 71	0.72	0.10	(0.51, 0.92)	<0.001	
Mode of bilateral stimulation	7.79	2, 71				<0.001	0.16
Eye movement–Tapping			−0.24	0.06	(−0.36, −0.12)	<0.001	
Eye movement–Eye movement & Tapping			−0.19	0.10	(−0.40, 0.01)	0.066	
Tapping–Eye movement & Tapping			0.05	0.11	(−0.16, 0.26)	0.655	
General work experience	1.37	1, 71	0.01	0.01	(−0.01, 0.02)	0.246	0.02
EMDR experience	0.07	1, 71	0.00	0.01	(−0.02, 0.01)	0.793	0.00

*CI, confidence interval. Overall model statistic: F(4,71)=3.93, p=0.006, R^2^ =18.1%*.

Linear *post hoc* contrasts replicated that eye movements enabled to reduce the SUD significantly stronger than tapping (23.8%, *p*_Holm_<0.001), while there was no difference between eye movements and combined eye movements and tapping (*p*_Holm_=0.166) and tapping and combined eye movements and tapping (*p*_Holm_=0.649).

### Therapists’ Attitudes Towards eEMDR

Therapists most frequently reported that unstable internet connection either on patient or therapist side to be a barrier to implement eEMDR (37.5%). Several therapists also indicated that their patients refused online-based therapy (31.3%). Some therapists considered lack of personal contact (28.1%) and missing withdrawal options for the patients in their flat (21.9%) as barriers. 6.3% of the therapists considered their own media and technology skills or their patients’ cognitive or sensory limitations as barriers. Interestingly, no therapist indicated high impulsivity of patients as a barrier for online therapy.

Furthermore, therapists were asked to report advantages and disadvantages of eEMDR sessions compared to face-to-face EMDR sessions as free text. Qualitative statements about advantages and disadvantages were reviewed and combined to superior categories. Results and frequencies of reported disadvantages can be seen in Table 5. Answers to the question concerning therapists’ perceived advantages of eEMDR sessions compared to face-to-face EMDR sessions were clustered into five categories as displayed in Table 6.

**Table 5:** Frequencies of perceived disadvantages in eEMDR sessions compared to face-to-face EMDR sessions.

	Frequency (%)^a^
Difficulties in detecting facial expression/gestures/eye movements	8 (34.8)
Difficulties with technology and internet connection	4 (17.4)
Difficulties concerning the EMDR process	4 (17.4)
Missing personal contact	3 (13)
Difficulties with creating therapeutic empathy	3 (13)
Negative implications of domestic surroundings	2 (8.7)

a *Relative numbers refer to therapists who returned eEMDR session protocols (N=23)*.

*Qualitative statements were reviewed and combined to superior categories*.

**Table 6:** Frequencies of perceived improvements in eEMDR sessions compared to face-to-face EMDR sessions.

	Frequency (%)^a^
Better patient’s focus and engagement in the therapy process	4 (17.4)
Positive implications of domestic surroundings	2 (8.7)
Easier for patients to allow emotions	1 (4.3)
Improvements in detecting facial expression (through the possibility to focus on the screen)	1 (4.3)
Flexibility concerning time and place	1 (4.3)

a *Relative numbers refer to therapists who returned eEMDR session protocols (N=23)*.

*Qualitative statements were reviewed and combined to superior categories*.

Difficulties with internet connection and dissociating patients were frequent answers concerning perceived disadvantages of eEMDR sessions. We therefore took a closer look at the actual connectivity issues and occurrence of dissociation during eEMDR sessions. Results showed that in 13.7% of the sessions, the connection was interrupted. Nevertheless, a SUD decrease of 68.3% could be accomplished in those sessions, and half of those sessions could be ended completely. Dissociations occurred in patients in 11.8% of the sessions. In those sessions, the mean relative SUD decrease was 53%, and one-third of those sessions could be ended as complete sessions.

Vast majority (87%) of the therapists stated, they would continue offering eEMDR beyond the COVID-19 pandemic. Conversely, 13% of the therapists considered *not* to offer eEMDR post pandemic. Qualitative statements indicated, the reasons against and in favor of continuing to offer eEMDR coincided with its perceived disadvantages and advantages. Besides, some therapists suggested eEMDR could be inappropriate for certain patients, including patients with dissociation and severe mental disease status. With appropriate patient groups, however, therapists stated they consider continuing to offer eEMDR beyond the COVID-19 pandemic.

In roughly half of the session protocols, the therapists provided information about their ideas and wishes for adjustments and improvements with respect to the future use of eEMDR beyond the COVID-19 pandemic. Answers were clustered into four categories as shown in Table 7. Mainly, the therapists emphasized the need of technical improvements in terms of a more reliable internet connection.

**Table 7:** Adjustments and improvements to eEMDR for further use of eEMDR beyond the pandemic.

Adjustment/improvement	Frequency (%)^a^
Reliable and stable internet connection	30 (29.4)
Appropriate framework (permission to work from home, permission from health insurances, technical equipment, surcharge)	12 (11.8)
Quality of platforms/programs (certified, safe, for free)	11 (10.8)
Reduce personal expectations	1 (1)

a *Relative numbers refer to all returned eEMDR session protocols (N=102)*.

*Qualitative statements were reviewed and combined to superior categories*.

## Discussion

Responses from therapists and the patients’ relative SUD decrease suggest eEMDR sessions can be an efficient and practically applicable alternative for face-to-face EMDR. Throughout eEMDR sessions examined in this study, therapist ratings of adherence, confrontation, grounding, and process were consistently positive, and the quality of more than 90% of the sessions was rated as *good* or *very good*. In this study, the most important indicator of effectiveness was the extent to which eEMDR enabled to reduce the patient-rated SUD from beginning to end of the session. According to Kim et al. (2008) the SUD score is an important and valid number for therapists to evaluate the treatment process. We calculated a relative SUD decrease to take into account the varying SUD ratings at session beginning. In sessions with available SUD ratings, the SUD rating dropped by 73.1% on average. This reduction is comparable to SUD reductions in other studies with clinical samples (Shapiro, 1989; Wilson et al., 1995; Ironson et al., 2002).

Importantly, the SUD decrease was independent from the therapists’ professional experience or their EMDR experience. The question whether therapists’ experience influences treatment outcome has been debated since the origins of psychotherapy (Goldberg et al., 2016) and findings are inconsistent (Propst et al., 1994; Tschuschke et al., 2015; Goldberg et al., 2016). Our results corroborate previous findings showing that treatment outcomes are widely independent from the therapists’ experience (Propst et al., 1994). These results are encouraging especially for newly licensed therapists and newly EMDR-trained therapists. Furthermore, the SUD decrease was independent from the age and sex of both therapists as well as patients. Our results therefore dispel negative expectations based on the preconception that older people might be less tech-savvy and insecure in the use of new media. Importantly, our results clearly emphasize that older therapists and patients should not have any reservations about attempting eEMDR.

In our study, the most relevant predictor of effective SUD reduction was the mode of bilateral stimulation. Administering bilateral stimulation *via* eye movements enabled significantly stronger decrease in the patients’ SUD than the use of tapping or a combination of eye movements and tapping. These results were also corroborated in a regression analysis when controlling the therapists’ professional and EMDR experience. With regard to the debate on the effectiveness of different modes of bilateral stimulation (Shapiro, 2013), our findings suggest that eye movements are at least more effective in reducing SUD ratings than tapping in eEMDR settings.

Within qualitative therapist statements, the most frequently named disadvantages of eEMDR compared to face-to-face EMDR were difficulties in detecting facial expressions, gestures, and eye movements on the screen. Other frequently referred challenges include difficulties with technology and connectivity issues and difficulties concerning the EMDR process (e.g., necessary adjustments to stabilization and reorientation procedure). However, technology and connectivity issues are concerns that can be resolved through further network expansion throughout the country (especially in rural areas) and also by providing adequate technical equipment. According to the therapists, one of the advantages of eEMDR compared to face-to-face EMDR is that the patients would better focus on and engage in the therapy process. Some therapists also observed positive implications of the patients’ domestic surroundings (e.g., patients felt more at ease when they were in their familiar environment). The barriers mentioned by therapists were not of therapeutic concerns, but rather related to the bureaucratic and technical framework of eEMDR. Importantly, more than 80% of the therapists participating in our study indicated they consider offering eEMDR beyond the COVID-19 pandemic, and this underscores the potential of eEMDR.

### Limitations

The present study is of explorative nature and it focused on the outcome from a single eEMDR session instead of an entire EMDR therapy. In addition, there was no comparison group to compare the effects of eEMDR to a waitlist-control group, face-to-face EMDR, or other internet-delivered therapy methods. Although this was not the intention of our exploratory study, randomized clinical trials are clearly warranted to ascertain the effectiveness of eEMDR as compared to its face-to-face version and other therapy approaches.

Notably, only 8% of the contacted therapists decided to participate, thus representing a selective and potentially biased subgroup of all therapists. The low response rate may be attributed to the restraint to offer EMDR *via* videoconference but also to a lack of time and capacity considering the strenuous workload for therapists due to the pandemic situation.

The questionnaire asked therapists regarding their attitude and experiences with eEMDR. There could be therapist bias due to social desirability, especially regarding effectiveness measures as they may have chosen not to report on those eEMDR sessions which were assessed as ineffective. However, in the sessions where the standard protocol was applied, nearly half of the sessions ended incompletely, thus dispelling the presumption that therapists selectively returned protocols of only effective sessions. Furthermore, patient-rated SUD values might be also biased by social desirability.

Future research could assess the experiences and compliance of patients participating in eEMDR, as this will provide additional insights for improving effectiveness of eEMDR. Furthermore, studies could investigate which patients benefit most from eEMDR formats and conversely, which diagnoses, or patient characteristics contraindicate the use of eEMDR.

## Conclusion

This study was one of the first to address the effectiveness of eEMDR by asking EMDR-therapists about their experiences with this treatment format. Our results show eEMDR as an effective and viable alternative to face-to-face EMDR. Especially the high SUD decrease in eEMDR sessions, an important indicator of treatment outcome was very promising. Thus, the results help to dispel doubts regarding the feasibility and appropriateness of EMDR *via* videoconference (Gibson et al., 2009; DPtV, 2020).

Therapists can consider conducting eEMDR even beyond the COVID-19 pandemic situation, as perceived impediments and disadvantages were mainly related to the bureaucratic and technical framework of eEMDR. However, with certain adjustments to the framework it is feasible to overcome these barriers. Thus, eEMDR has the potential to not only be a temporary solution during the pandemic but to become an integral part of everyday therapy.

## Data Availability Statement

The original contributions presented in the study are included in the article/supplementary material, further inquiries can be directed to the corresponding authors.

## Ethics Statement

Ethical review and approval was not required for the study on human participants in accordance with the local legislation and institutional requirements. Written informed consent from the patients/ participants or patients/participants legal guardian/next of kin was not required to participate in this study in accordance with the national legislation and the institutional requirements.

## Author Contributions

VT conceptualized the study, obtained ethical approval, and recruited therapists. CM wrote the manuscript under supervision of VT and AB, and performed statisical analysis together with LM under supervision of AB. AH provided expertise and feedback and recruited therapists. ML provided data. SV and RR edited the manuscript. I-TK supervised the study, revised and edited the manuscript. All authors contributed to the article and approved the submitted version.

## Conflict of Interest

The authors declare that the research was conducted in the absence of any commercial or financial relationships that could be construed as a potential conflict of interest.

## Publisher’s Note

All claims expressed in this article are solely those of the authors and do not necessarily represent those of their affiliated organizations, or those of the publisher, the editors and the reviewers. Any product that may be evaluated in this article, or claim that may be made by its manufacturer, is not guaranteed or endorsed by the publisher.
